# Supplementary material for: Scaled, high fidelity electrophysiological, morphological, and transcriptomic cell characterization
Source: eLife. 2021 Aug 13;10:e65482. doi: 10.7554/eLife.65482 (PMC8428855; doi:10.7554/eLife.65482)
Supplement: Supplementary file 1. — The purpose of this table is to list the different parameters and purpose for each stage within the Patch-seq protocols, the metrics measured, the subsequent data and range of data obtained. The criteria that the Allen Institute uses for Patch-seq can be adopted, relaxed, or disregarded. 1 Electrophysiology, morphology and transcriptomic parameters and metrics can also be found in the technical white papers at http://celltypes.brain-map.org. [file elife-65482-supp1.docx]

| **Name** | **Stage** | **Purpose** | **Measurement details** | **Measurement frequency** | **Measurement Quan/Qual** | **Data range** | **Allen Institute criteria** | **Relevant figure panels** |
| --- | --- | --- | --- | --- | --- | --- | --- | --- |
| Seal resistance | Patch clamp recording | Maximize the chance of a stable recording session | During the patch clamp process, after tight seal formation and before break-in, 10 ms, 10 mV voltage clamp deflection. Resistance measured at end of sweep. | Once per cell, at the beginning of the ephys experiment | Quantitative | > 0 MΩ | > 1000 MΩ |  |
| Access resistance (Ra) | Patch clamp recording | Maximize the chance of a stable recording session | Ra is measured upon break-in using a (10 ms, 10 mV) voltage clamp deflection. A (10 ms, 50 pA) "test pulse" is also inserted before each current clamp sweep to monitor changes in Ra. | At the beginning of the ephys experiment and each sweep | Quantitative | > 0 MΩ | < 20 MΩ |  |
| Resting membrane potential (RMP) | Patch clamp recording | Maximize the chance of a stable recording session and confirm the cell is healthy | RMP is observed upon switching to current clamp. At least one sweep is acquired at RMP before applying any bias current. | Once per cell, at the beginning of the ephys experiment | Quantitative | < 0 mV | Between -60 and -80 mV (dependent on cell type) | Fig 3,  Fig 3-Sup Fig 3 |
| Membrane voltage (Vm) | Patch clamp recording | Ensure the reliability of data collected and monitor for any aberrations | Vm at the start of the sweep is compared to Vm at the end of the sweep to calculate delta Vm. | At the beginning and end of each sweep | Quantitative | < 0 mV | Delta Vm must be within +/- 1 mV | Fig 3,  Fig 3-Sup Fig 3 |
| Bias current | Patch clamp recording | Ensure the cell remains healthy and the RMP is consistent through the duration of the experiment | The amount of bias current applied to hold the cell at the initial RMP | At the beginning of each sweep | Quantitative | +/- 500 pA | +/- 100 pA | Fig 3,  Fig 3-Sup Fig 3 |
| AP amplitude | Patch clamp recording | Ensure the reliability of data collected and monitor for any aberrations | Height of the AP | During each firing sweep | Quantitative | > 0 mV | AP must cross 0 mV | Fig 3,  Fig 3-Sup Fig 3 |
| Nucleus Capture | Patch clamp recording | Extraction of the nucleus is key to optimal transcriptomic content | Visual confirmation of nucleus attached to tip of pipette | Once per cell, at the conclusion of withdrawing pipette from cell and slice | Qualitative | nucleus+ or nucleus- | Collection of nucleus+ samples only | Fig 2 |
| end-seal resistance (endR) | Patch clamp recording | The strength of the seal around the nucleus (or tip of pipette) after retraction correlates with successful biocytin fill | At the conclusion of the patch-clamp recording, a 10 ms, 10 mV voltage clamp deflection is administered during nucleus extraction and retraction periods. | Continuous, every 40 ms, until the pipette is removed from the tissue. | Quantitative | > 0 MΩ | Collection of samples with > 100 MΩ | Fig 2,  Fig 5 |
| **Electrophysiology success defined as: Passing sweeps and QC measured in MIES** | | | | | | | | |
| Fragment/Bioanalyzer traces | cDNA processing | Evaluate electrophoretograms for quality and quantity of amplified cDNA | Evaluation of the shape and distribution of the trace. | Once per sample | Qualitative | NA | All samples proceed to sequencing |  |
| cDNA quality | cDNA processing | To evaluate distribution ( base pair length) of the cDNA | Obtained from the fragment/bioanlyzer trace and defined as the percent of high base pair material, with 400 base pair as the demarcation between high and low | Once per sample | Quantitative | 0-100% | All samples proceed to sequencing | Fig 5-Sup Fig 2 |
| cDNA quantity | cDNA processing | Quanitification of the total amont of cDNA | Obtained from the fragment/bioanlyzer trace (or from Picogreen analyses) and is total material in nanograms | Once per sample | Quantitative | > 0 ng | All samples proceed to sequencing | Fig 5-Sup Fig 2 |
| Genes detected | Sequencing | To evaluate the total number of genes measured obtained from a patch-seq recording | Next generation sequencing is used to calculate the total number of genes obtained from each sample. | Once per sample | Quantitative | 0-15,000 genes | All samples evaluated | Fig 5-Sup Fig 2 |
| Normalized marker sum (NMS) | Sequencing | To evaluate the specificity of the genes detected from a patch-seq recording | A ratio of the average expression of 'on' marker genes. | Once per sample | Quantitative | 0-1.2 | NMS > 0.4 | Fig 4, Fig 5  Fig 5-Sup Fig 2 |
| **Transcriptomic success defined as: NMS > 0.4** | | | | | | | | |
| Biocytin fill | Morphology | Identification of layer and region of patched cell | Identification of filled biocytin cell or biocytin plume under 20x magnification. | Once per cell | Qualitative | NA | NA | Fig 6,  Fig 6-Sup Fig 1, Fig 6-Sup Fig 4 |
| Morphology call | Morphology | To evaluate the quality of the biocytin fill and morphology of the patched neuron, ultimately deciding which cells move on to be reconstructed. | Single cells are processed to view their biocytin fills under 20x and/or 63x magnification. | Once per cell | Qualitative | Failed fill - no visible soma or processes; Insufficient axon - for inhibitory neurons only, visible dendrites but no visible axon; Medium quality - visible soma and incomplete fill of dendrites and/or axon; High quality - visible soma, dendrites and axon | Only 'High quality' cells proceed to 3D digital reconstruction | Fig 6,  Fig 6-Sup Fig 1,  Fig 6-Sup Fig 4 |
| **Morphology success defined as: morphological call = Medium or High quality** | | | | | | | | |
